# Pentoxifylline vaginal gel improves sperm motility and vitality in asthenozoospermic males: A randomized, blinded, *in vitro* study

**DOI:** 10.3389/fphar.2025.1669045

**Published:** 2025-11-26

**Authors:** Cristina Trilla, Lidia Navarro, Mariona Rius, Juan José Espinós

**Affiliations:** 1 Clínica Fertty, Barcelona, Spain; 2 Prokrea BCN S.L., Barcelona, Spain; 3 Department of Obstetrics and Gynecology, Hospital de la Santa Creu, Barcelona, Spain; 4 Faculty of Medicine, Autonomous University of Barcelona, Barcelona, Spain; 5 Spanish Network in Maternal, Neonatal, Child and Developmental Health Research (RICORS-SAMID, RD24/0013/0001), Instituto de Salud Carlos III, Madrid, Spain

**Keywords:** asthenozoospermia, pentoxifylline, sperm motility, sperm vitality, *in vitro* study, male infertility, vaginal gel

## Abstract

**Introduction:**

In this study, we aimed to evaluate the *in vitro* effects of a 4% pentoxifylline-containing vaginal gel (PKB171 4%) on sperm motility and vitality in samples from asthenozoospermic males, compared with its placebo vaginal gel.

**Methods:**

This was a prospective, blinded, placebo-controlled study conducted at Fertty Clinic, Barcelona, Spain. Thirty-two semen samples from men with asthenozoospermia were randomized for treatment with either PKB171 gel containing 4% of pentoxifylline or a PKB171 placebo gel. Sperm motility and vitality were assessed at baseline and at three points following exposure: immediately (T0), after 45 min (T45), and after 180 min (T180). The evolution of sperm motility and vitality over time after exposure was compared between the groups.

**Results:**

At T45, the group treated with PKB171 4% showed an increase in progressive motility compared with T0. Progressive motility was significantly higher in the PKB171 4% group than in the placebo group (35.3% vs. 28.9%, p = 0.035). Compared to baseline, the PKB171 4% group experienced only a 1% decline in progressive motility, whereas the placebo group exhibited a 10.8% reduction (p = 0.011). Vitality was also more preserved in the PKB171 4% group, with significantly lower vitality decline at T45 and T180 than in the placebo group. Although motility and vitality decreased over time in both groups, the decline was consistently less severe in samples treated with the PKB171 4% gel.

**Discussion:**

The PKB171 4% gel significantly improves progressive motility and preserves sperm vitality in sperm samples from asthenozoospermic males. These promising *in vitro* findings require further clinical investigation to confirm the potential benefits in infertile couples with male infertility.

## 1 Introduction

Infertility affects approximately 10%–20% of couples worldwide, with male factor infertility accounting for up to 30% of cases, either as an isolated cause or in combination with female factors (Sun et al., 2019; Mazzilli et al., 2023). Despite this significant contribution, clinical and research efforts continue to focus disproportionately on female factors, often leaving male infertility underexplored and undertreated.

The standard assessment of male fertility typically begins with a semen analysis based on the World Health Organization (WHO) reference criteria. According to the sixth edition of the WHO Laboratory Manual, normal semen should have a volume ≥1.4 mL, sperm concentration ≥16 million/mL, progressive motility ≥30%, and normal morphology ≥4% (World Health Organization, 2021). Among these parameters, reduced sperm motility—asthenozoospermia—is considered one of the most significant predictors of poor outcomes in assisted reproductive technologies (ART) (Villani et al., 2022).

Pentoxifylline is a methylxanthine-derived hemorheological agent primarily used to improve microcirculatory blood flow by increasing erythrocyte deformability and reducing blood viscosity (Ward and Clissold, 1987). Its pharmacologic action as a nonselective phosphodiesterase inhibitor increases intracellular cyclic adenosine monophosphate (cAMP) levels, which has been associated with enhanced sperm motility and viability (Negri et al., 1996). Pentoxifylline has been studied both as an oral therapy in men and as an *in vitro* additive treatment for semen samples, with prior investigations suggesting its potential to enhance sperm motility in cases of asthenozoospermia (Yunes et al., 2005; Mehrannnia, 2009; Merino et al., 1997; Kovacic et al., 2006). Moreover, some clinical studies have reported improved pregnancy rates when pentoxifylline was used in semen preparation prior to intrauterine insemination (IUI) or *in vitro* fertilization (IVF) (Shen et al., 1991; Yovich et al., 1988).

Despite these promising findings, the design of previous studies often had several methodological shortcomings, including inadequate control groups, lack of randomization, and absence of blinding. Additionally, contemporary laboratory techniques now allow for more accurate and reproducible assessments of sperm motility and vitality.

Building on this pharmacological background, a novel aqueous vaginal formulation of pentoxifylline (PKB171 4%) was developed by Prokrea BCN S.L. (Barcelona, Spain) to enable localized drug delivery at the site of semen deposition. Contemporary reviews highlight the pharmacological advantages of vaginal delivery, including high local concentrations, avoidance of first-pass metabolism, and lower systemic side effects (Sanchez Armengol et al., 2025). These pharmacological properties provide a strong rationale for exploring vaginal pentoxifylline formulations as a novel, locally acting approach to enhance sperm function and support natural conception in selected infertility contexts. The PKB171 4% gel was designed according to the Spanish National Formulary standards for neutral gels, with the pH adjusted for vaginal compatibility and validated antimicrobial preservation. Preclinical pharmacokinetic and safety studies demonstrated favorable local tolerance and minimal systemic exposure after repeated vaginal administration in animal models (Non-Clinical Study 1562, Final CSR and Non-Clinical Study 1275, Final CSR). Subsequently, a phase-I clinical trial (PKB171-01) in healthy women confirmed the local safety and tolerability of the gel, with no serious adverse events and only mild, transient reactions reported (Ballester et al., 2020).

Given the promising preclinical and safety data, we conducted a prospective, randomized, blinded, placebo-controlled *in vitro* study to investigate whether the observed motility-enhancing effects of pentoxifylline on semen samples could be replicated under controlled laboratory conditions using this new formulation. We hypothesized that PKB171 4% would improve progressive sperm motility and help preserve vitality compared with the placebo.

## 2 Methods

This was a placebo-controlled, randomized *in vitro* study conducted under blinded laboratory conditions between March and July 2024 at Fertty Clinic, Barcelona, Spain, which is an accredited fertility center. The study was approved by the Ethics Committee of Hospital del Mar (Barcelona) (approval No. 2023/11309/I). Written informed consent was obtained from all participating subjects prior to their inclusion in the study.

Male patients aged 18–50 years and undergoing fertility evaluation or treatment were invited to participate if they met the following inclusion criteria: body mass index (BMI) between 18 and 32, semen analysis demonstrating asthenozoospermia defined as total motility <40%, with vitality ≥45%, morphology ≥2%, and semen volume ≥1 mL. Sexual abstinence of 2–5 days was required before sample collection.

The exclusion criteria included azoospermia, seminal infections, varicocele, testicular trauma, prior vasectomy reversal, hydrocele, undescended testicles, and a history of exposure to radiation or gonadotoxic chemicals.

### 2.1 Sample collection and semen analysis

Each eligible donor contributed a single semen sample, which served as an independent experimental unit. Samples were not divided or aliquoted for parallel testing. Semen samples were collected via masturbation in sterile containers and allowed to liquefy at room temperature for 30 min. After liquefaction, basal semen analysis was carried out following the WHO 2021 guidelines (World Health Organization, 2021). All assessments were performed by the same experienced biologist (L.N.). The volume, concentration, motility (types A–D, %), morphology (%), and vitality (%) were determined. Seminal volume was determined with a graduated serological pipette, and sperm concentration was assessed by the conventional method using a Makler counting chamber (Sefi Medical Instruments, Israel) and expressed in millions/mL. The sperm motility was assessed in at least 100 sperm and expressed as the percent of motile sperm (the sum of rapid progression plus slow progression sperm). Vitality, expressed as percent, was determined using Eosin–Nigrosin staining, and morphology was evaluated using cresyl violet acetate and new methylene blue staining (Testsimplets®, Waldeck).

Following liquefaction and baseline evaluation, each semen sample was randomized (1:1) for treatment with the PKB171 gel without pentoxifylline (placebo or gel A) or PKB171 gel containing 4% pentoxifylline (gel B). The PKB171 gel containing 4% pentoxifylline is a patented gel [US patent number US 9,801,885 B2; European patent number EP2724716B1; Patent Cooperation Treaty (PCT) number WO/2012/175775A1)]. The gels were identical in appearance and coded by the batch number. Randomization was performed using RandList software v1.2. Randomization codes were generated by an independent investigator not involved in the laboratory assessments. Laboratory personnel performing motility and vitality assessments, as well as data analysts, were blinded to the allocation codes until the completion of statistical analysis. As this was an *in vitro* study, no patient-level treatment was administered, and semen donors were not aware of the group assignment.

After baseline analysis, 0.1 g of either gel was diluted in 1.9 mL of the wash medium (PureSperm® Wash, Nidacon). Then, 0.5 mL of this dilution was mixed with 0.5 mL of the participant’s semen (1:1 ratio) and incubated at 37 °C. Samples were analyzed at three points: immediately after mixing (T0), 45 min post-exposure (T45), and 180 min post-exposure (T180). The 45-min interval was selected in alignment with the World Health Organization (2021) guidelines, which recommend semen evaluation within 30 min–60 min after liquefaction, and to capture the expected early pharmacodynamic peak effect of pentoxifylline on motility (McKinney et al., 1994). The 180-min time-point was chosen to assess the persistence or decay of the effect over a clinically relevant period, and it also reflected the typical time frame used for sperm preparation in assisted reproduction laboratories (Punjabi et al., 2021).

### 2.2 Outcomes

The primary outcome was the change in progressive motility (%) at T45 compared to baseline between the two groups. Secondary outcomes included the change in sperm vitality at T45 and T180, and the comparison of motility (%) and vitality (%) among T0, T45, and T180 within and between the groups.

### 2.3 Sample size calculation and statistical analysis

A minimum of 32 participants (16 per group) was required to detect a ≥5% difference in progressive motility between groups with 80% power and α = 0.05. This accounted for an expected standard deviation of 6%–10% in motility parameters.

Data were analyzed using SPSS v29.0 (IBM Corp., Armonk, NY). Normality of distribution was verified with the Kolmogorov–Smirnov test. Continuous variables were expressed as the mean ± standard deviation (SD). Group comparisons were made using Student’s t-test or the Mann–Whitney U test as appropriate. Longitudinal comparisons were performed using two-way repeated-measures ANOVA, with treatment (PKB171 4% vs. placebo) as the between-subject factor and time as the within-subject factor. The assumption of sphericity was not formally tested; however, given that only three time points were analyzed, the potential impact of sphericity violations on the results is expected to be minimal. A p-value ≤0.05 was considered statistically significant.

## 3 Results

**FIGURE 1:**
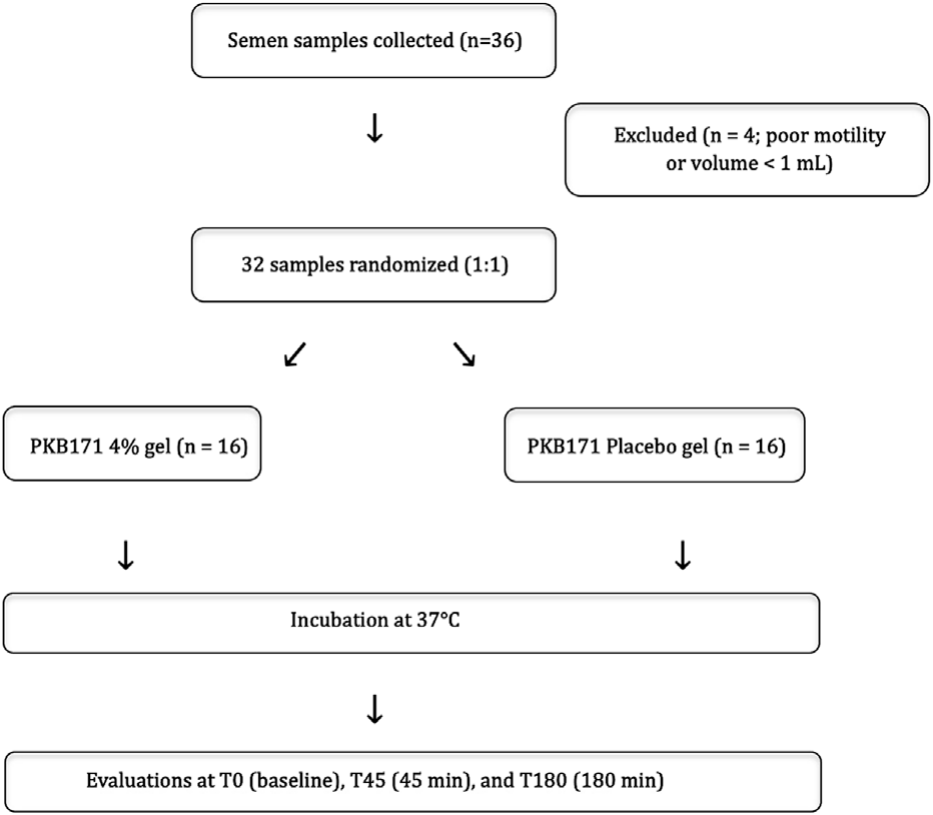
Experimental workflow and sample allocation. Flowchart representation of the *in vitro* experimental workflow illustrating the selection, randomization, and analysis of semen samples allocated to PKB171 4% gel or placebo. Motility and vitality were evaluated at baseline (T0), 45 minutes (T45), and 180 minutes (T180).

A total of 36 patients were screened, and 32 of them fulfilled all inclusion criteria and were enrolled (n = 16 per group). The experimental workflow, including sample selection, randomization, and timing of motility and vitality assessments, is summarized in Figure 1. Table 1 summarizes the baseline characteristics and semen analysis of the study participants. Demographic, medical, and reproductive characteristics were similar between the groups, except for a non-statistically significant proportion of live children in the pentoxifylline group (8% vs. 2%, p = 0.054). No statistically significant differences in baseline semen parameters were observed between groups, except for vitality, which was slightly higher in the pentoxifylline group (70.1% vs. 64.7%, p = 0.017). All participants were diagnosed with asthenozoospermia.

**TABLE 1:** Comparison of the baseline characteristics of the study participants between the PKB171 placebo gel (n = 16) and the PKB171 4% gel (n = 16).

	PKB171 placebo gel N = 16	PKB171 4% gel N = 16	*p*-value
Patient characteristics^a^			
Age (years)	38.07 (7.68)	37.32 (5.08)	0.748
BMI (kg/m^2^)	25.73 (3.33)	24.86 (3.24)	0.461
Medical history^b^			
Diabetes	0 (0)	0 (0)	>0.999
Hypertension	6.3 (1)	0 (0)	>0.999
Thyroid	0 (0)	6.3 (1)	>0.999
Other	0 (0)	12.5 (2)	0.484
Reproductive history^b^			
Previous pregnancies	50 (8)	50 (8)	>0.999
Live children	12.5 (2)	50 (8)	0.054
Children born after ART	12.5 (2)	37.5 (6)	0.220
Baseline seminal analysis^a^			
Volume (mL)	2.88 (0.98)	3.16 (1.37)	0.521
Concentration (M/mL)	47.53 (29.67)	49.08 (40.68)	0.903
Progressive (M/mL)	16.83 (11.12)	18.25 (16.83)	0.781
Nonprogressive (M/mL)	1.45 (0.69)	1.58 (0.78)	0.634
Immotile (M/mL)	29.23 (18.62)	29.25 (23.52)	>0.999
Progressive (%)	35.09 (5.72)	35.30 (4.92)	0.915
Nonprogressive (%)	4.10 (2.72)	4.46 (3.15)	0.729
Immotile (%)	60.81 (6.63)	60.24 (5.71)	0.797
Normal forms (%)	3.06 (0.85)	3.25 (0.85)	0.540
Vitality	64.75 (5.97)	70.13 (6.09)	0.017

^a^ Data are presented as the mean (SD).

^b^ Data are presented as n (%).

BMI, body mass index; hypertension, high blood pressure; ART, assisted reproduction treatment; M/mL, millions per milliliter.

Results regarding the comparison of sperm parameters over time between groups are presented in Tables 2–4. In samples allocated to group A (gel PKB171 placebo), progressive motility and vitality decreased steadily over time. At T45, the mean progressive motility dropped from 30.82% to 28.91%, and it further declined to 25.05% at T180. Vitality declined from 61.5% at T0 to 53% at T180. In contrast, samples allocated to group B (Gel PKB171 4%) showed an initial improvement in motility at T45, with progressive motility increasing from 32.03% to 35.33%. Vitality remained relatively stable (63.62%–62.0%). At T180, both parameters declined slightly, but they remained higher than those in the placebo group (progressive motility 29.24% vs. 25.05%, vitality 57.44% vs. 53.0%).

**TABLE 2:** Comparison of sperm analysis at time 0 between the PKB171 placebo gel (n = 16) and the PKB171 4% gel (n = 16).

	PKB171 placebo gel N = 16	PKB171 4% gel N = 16	Mean difference (95% CI)	*p*-value
Concentration (M/mL)	24.60 (14.94)	25.84 (22.67)	1.24 (−12.06–14.54)	0.856
Progressive (M/mL)	7.86 (5.48)	9.11 (9.39)	1.25 (−4.08–6.85)	0.649
Nonprogressive (M/mL)	1.20 0.61)	1.29 (0.68)	0.09 (−0.36–0.54)	0.684
Immotile (M/mL)	15.54 (19.38)	15.44 (12.93)	−0.10 (−11.53–11.33)	0.980
Progressive (%)	30.82 (7.09)	32.03 (7.01)	1.21 (−3.67–6.09)	0.630
Nonprogressive (%)	6.12 (3.22)	5.99 (2.28)	−0.13 (−2.07–1.81)	0.902
Immotile (%)	63.06 (7.74)	61.97 (7.06)	−1.09 (−6.22–4.04)	0.680
Vitality (%)	61.50 (5.33)	63.62 (6.56)	2.12 (−2.02–6.26)	0.323

Data are presented as the mean (SD).

M/mL, millions per milliliter; CI, confidence interval.

**TABLE 3:** Comparison of sperm analysis after 45 min of exposure between the PKB171 placebo gel (n = 16) and the PKB171 4% gel (n = 16).

	PKB171 placebo gel N = 16	PKB171 4% gel N = 16	Mean difference (95% CI)	*p*-value
Concentration (M/mL)	25.07 (14.70)	25.94 (22.05)	0.87 (−12.1–13.9)	0.897
Progressive (M/mL)	7.64 (5.41)	10.04 (10.57)	2.40 (−3.42–8.22)	0.424
Nonprogressive (M/mL)	1.45 (0.77)	1.13 (0.67)	−0.32 (−0.82–0.18)	0.223
Immotile (M/mL)	15.99 (9.19)	14.77 (11.77)	−1.22 (−8.53–6.09)	0.746
Progressive (%)	28.91 (7.25)	35.33 (9.13)	6.42 (0.60–12.3)	0.035
Nonprogressive (%)	7.27 (4.21)	5.26 (3.43)	−2.01 (−4.68–0.66)	0.154
Immotile (%)	63.82 (7.29)	59.41 (10.26)	−4.41 (−10.58–1.76)	0.171
Vitality (%)	57.63 (7.27)	62.00 (6.43)	4.37 (−0.39–9.13)	0.082
Difference from T0				
Progressive (M/mL)	−1.91 (3.18)	3.30 (5.60)	5.21 (2.06–8.36)	0.004
Progressive (%)	−3.48 (5.31)	4.68 (8.56)	8.16 (3.22–13.10)	0.003
Vitality (%)	−3.88 (6.61)	−1.63 (1.02)	2.25 (−1.03–5.53)	0.028

Data are presented as the mean (SD).

M/mL, millions per milliliter; CI, confidence interval.

**TABLE 4:** Comparison of sperm analysis after 180 min of exposure between the PKB171 placebo gel (n = 16) and the PKB171 4% gel (n = 16).

	PKB171 placebo gel N = 16	PKB171 4% gel N = 16	Mean difference (95% CI)	*p*-value
Concentration (M/mL)	24.86 (14.59)	25.68 (21.81)	0.82 (−12.04–13.68)	0.902
Progressive (M/mL)	6.72 (4.92)	8.14 (7.91)	1.42 (−3.15–6.99)	0.547
Nonprogressive (M/mL)	1.25 (0.65)	1.41 (0.82)	0.16 (−0.35–0.67)	0.554
Immotile (M/mL)	16.89 (9.74)	16.14 (13.58)	−0.75 (−9.94–7.44)	0.858
Progressive (%)	25.05 (9.55)	29.24 (7.31)	4.19 (−1.71–10.09)	0.173
Nonprogressive (%)	6.09 (2.97)	6.34 (3.11)	0.25 (−1.85–2.35)	0.812
Immotile (%)	68.86 (8.84)	64.41 (7.56)	−4.45 (−10.15–1.25)	0.136
Vitality (%)	53.00 (7.71)	57.44 (7.73)	4.44 (−0.91–9.79)	0.114
Difference from T0				
Progressive (M/mL)	−5.77 (6.41)	−2.78 (4.51)	2.99 (−0.85–6.83)	0.139
Progressive (%)	−13.39 (16.06)	−4.79 (8.05)	8.60 (−0.20–17.40)	0.065
Vitality (%)	−8.50 (4.18)	−6.19 (2.99)	2.31 (−0.20–4.82)	0.082

Data are presented as the mean (SD).

M/mL, millions per milliliter; CI, confidence interval.

At T45, the mean increase in progressive motility from T0 in the PKB171 group was +4.68% compared to a −3.48% decline in the placebo group (p = 0.003). At T180, progressive motility decreased in both groups compared to that in T0, but it remained higher in the PKB171 4% group, although this difference did not reach statistical significance (−4.79% vs. −13.39% from T0, p = 0.065).

**FIGURE 2:**
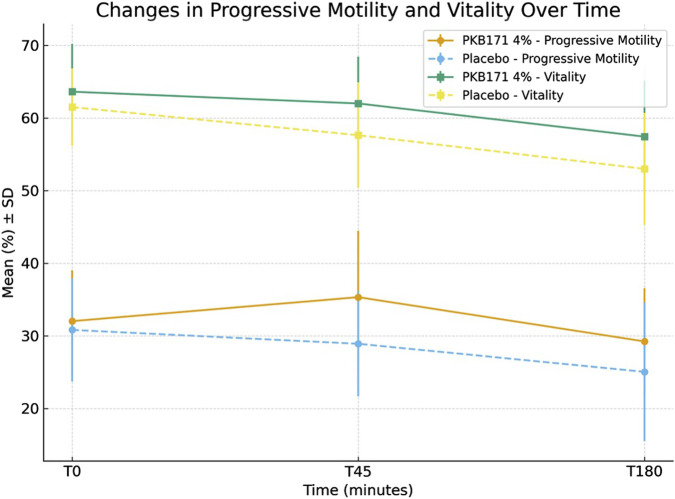
Changes in progressive motility and vitality over time (Figure 1). Changes in progressive motility and vitality over time in semen samples incubated with PKB171 4% (n=16) or placebo gels (n = 16). Data are presented as the mean (%) ± SD.

Regarding vitality, at T45, vitality decreased in both groups, but this decrease was more significant in the placebo group (−3.48% vs. −1.29%, p = 0.029). At T180, both groups showed a similar overall decline, although the reduction was less marked in the PKB171 4% group (−7.78% vs. −5.28%, p = 0.082). Changes in progressive motility and vitality over time are represented in Figure 2.

## 4 Discussion

In this *in vitro* study, we demonstrate that a 4% pentoxifylline gel (PKB171 4%) significantly improves progressive motility and helps to preserve the vitality of spermatozoa from asthenozoospermic males, particularly within 45 min of exposure. Although both treatment groups exhibited a time-dependent decline in sperm function, the pentoxifylline-treated group consistently showed a more favorable trajectory.

Our findings align with previous studies reporting improved motility with pentoxifylline, whether administered orally or used in sperm preparation media (Negri et al., 1996; Yunes et al., 2005; Mehrannnia, 2009; Merino et al., 1997; Shen et al., 1991). For instance, Negri et al. (1996) observed enhanced sperm motility and acrosome reaction following pentoxifylline treatment, suggesting its potential utility in assisted reproduction. However, previous studies have shown inconsistent results. Tournaye et al. found that indiscriminate use of pentoxifylline did not improve fertilization rates in poor fertilizing patients undergoing IVF (Tournaye et al., 1994). These discrepancies may be attributed to differences in study design, patient selection, and treatment protocols. Indeed, most of those earlier studies lacked rigorous methodological design, often being non-blinded or without placebo controls. Our study adds value by employing a randomized, blinded, placebo-controlled approach and using the current WHO criteria along with standardized staining and counting techniques to assess motility and vitality.

The mechanism of action of pentoxifylline in enhancing sperm motility is believed to be mediated through the inhibition of phosphodiesterases, leading to elevated intracellular cAMP levels and the subsequent activation of sperm motility pathways (Ward and Clissold, 1987; Yunes et al., 2005). The motility-enhancing effect of pentoxifylline is primarily mediated through the inhibition of phosphodiesterase activity, leading to an increase in intracellular cAMP levels. Elevated cAMP activates protein kinase A (PKA), which in turn promotes tyrosine phosphorylation of specific flagellar proteins. This signaling cascade has been closely associated with the acquisition of hyperactivated motility and capacitation-like changes in spermatozoa (Visconti et al., 1995; Yunes et al., 2005). In parallel, the antioxidant properties of pentoxifylline may contribute to preserving mitochondrial function and membrane integrity, thereby supporting sustained motility during incubation. Furthermore, pentoxifylline’s antioxidant properties may contribute to the maintenance of membrane integrity and sperm viability, which is particularly relevant in asthenozoospermic samples that may exhibit elevated oxidative stress. Studies have shown that an imbalance between reactive oxygen species (ROS) production and antioxidant products can impair sperm function, leading to decreased motility. For instance, high semen ROS levels are reported in 30%–80% of infertile men, suggesting a significant correlation between oxidative stress and sperm motility issues (Ribeiro et al., 2022).

In our study, the most notable difference in sperm motility was observed at 45 min of incubation, when the PKB171 4% group exhibited a 6.5% absolute advantage in progressive motility compared to placebo. Although this absolute increase in progressive motility may appear modest, even small relative improvements in the motile sperm fraction can be clinically meaningful. In assisted reproduction, success in fertilization is strongly dependent on the number of progressively motile spermatozoa available for insemination, as suggested by previous research (Lemmens et al., 2016; Van Voorhis et al., 2001). Therefore, the current findings provide a biologically plausible rationale for further clinical evaluation to determine whether the magnitude and duration of this motility improvement could impact fertilization outcomes. Moreover, the time-point at which this increase in motility is most pronounced may represent an optimal window for potential application in assisted reproductive technologies, such as intrauterine insemination (IUI) or *in vitro* fertilization (IVF), where sperm selection and timing are critical (Kuru Pekcan et al., 2018). It might also have implications in natural conception in couples affected by mild male factor; however, this has yet to be investigated. Finally, although the benefits of the PKB171 4% gel on sperm motility persisted at 180 min, the difference in progressive motility between the groups narrowed and did not reach statistical significance, suggesting a transient peak in efficacy.

Interestingly, vitality was better preserved in the PKB171 4% group, both immediately and over time. The protective effect on vitality may extend the functional lifespan of sperm, potentially offering a clinical advantage when sperm preparation or insemination is delayed. However, the observed differences in sperm vitality between the groups were statistically modest and should be interpreted with caution considering biological and analytical variability. The Eosin–Nigrosin staining technique primarily reflects plasma membrane integrity and, although standardized and recommended by the WHO for vitality assessment, has an inherent analytical variation (coefficient of variation typically 5%–10%) depending on the operator experience, sample homogeneity, and staining conditions (World Health Organization, 2021). Consequently, small changes in vitality may partly reflect measurement variability rather than true biological effects. Nevertheless, the consistent trend toward higher vitality values in the PKB171 4% group across time-points may suggest a mild stabilizing effect on membrane integrity, which is possibly related to the known antioxidant and rheological properties of pentoxifylline. This potential mechanism of action requires further investigation.

A major strength of this study is its robust design, including blinding, randomization, and controlled laboratory conditions. Importantly, in this study, we also addressed a clear translational gap, as few contemporary investigations have assessed the short-term *in vitro* effects of pentoxifylline in a controlled, reproducible manner using current reference guidelines. By doing so, our findings offer a modernized and replicable benchmark for future research on sperm activation strategies. Despite these strengths, several limitations must also be acknowledged. First, due to sample volume constraints inherent to most asthenozoospermic individuals, it was not possible to test both the treatment and placebo gels on aliquots from the same sample, which may have introduced interindividual variability. Second, although the sample size was sufficient to detect a 5% difference in progressive motility, larger studies may help confirm these results and detect smaller effects on vitality and other motility subtypes. Third, sphericity was not formally tested in the repeated-measures analysis. However, given that only three time-points were assessed, the potential effect of sphericity violations is likely minimal. Future studies including larger sample sizes and additional time-points could further confirm the robustness of these temporal findings. Finally, the clinical relevance of these findings, particularly their impact on fertilization and pregnancy rates, requires confirmation in prospective clinical trials.

This randomized, blinded, placebo-controlled *in vitro* study demonstrates that exposure to a 4% pentoxifylline gel (PKB171 4%) improves progressive motility and better preserves sperm vitality in samples from asthenozoospermic males. The effect was most pronounced at 45 min post-exposure, where a significant increase in progressive motility and a reduced decline in vitality were observed compared with the placebo group. Although both parameters declined over longer incubation periods, the deterioration was consistently less severe in the PKB171 4% group.

The present results provide a biological proof-of-concept for the motility-enhancing properties of the PKB171 4% gel observed under controlled *in vitro* conditions. Although these findings may suggest potential translational relevance, their clinical applicability remains speculative at this stage. From a translational perspective, a post-coitally applied vaginal gel offers a female-controlled, locally acting approach that has the potential of transiently increasing progressive motility of sperm while minimizing systemic exposure. Further well-designed, *in vivo*, prospective clinical trials are required to determine whether these *in vitro* effects translate into measurable improvements in fertilization or pregnancy outcomes, particularly in cases characterized by male infertility with reduced motility or borderline sperm parameters. To validate these *in vitro* findings, different complementary trial designs involving well-defined populations could be envisaged, including a timed-intercourse trial involving couples with mild male factor infertility and no major female factors, or an ART-adjacent randomized trial evaluating the same concentration of pentoxifylline as a processing additive during sperm preparation for IUI or conventional IVF. Thus, these results provide preliminary support for evaluating the potential benefit of a pentoxifylline gel as sperm-activating media in ART or natural conception.

## Data Availability

The raw data supporting the conclusions of this article will be made available by the authors upon reasonable request.
